# Cardiovascular CT in Bicuspid Aortic Valve Disease: A State-of-the-Art Narrative Review of Advances, Clinical Integration, and Future Directions

**DOI:** 10.3390/jcm15031268

**Published:** 2026-02-05

**Authors:** Muhammad Ali Jawed, Cagri Ayhan, Robert Byrne, Sandeep Singh Hothi, Sherif Sultan, Mark Spence, Osama Soliman

**Affiliations:** 1 Cardiovascular Research Institute (CVRI), D07 KWR1 Dublin, Ireland; 2 Mater Private Network, D07 KWR1 Dublin, Ireland; 3 Royal College of Surgeons in Ireland (RCSI), D02 YN77 Dublin, Ireland; 4 Department of Cardiology, Royal Wolverhampton Trust, Wolverhampton WV10 0QP, UK; 5 Western Vascular Institute, Department of Vascular and Endovascular Surgery, University Hospital Galway, University of Galway, H91 TK33 Galway, Ireland; 6 Department of Vascular Surgery and Endovascular Surgery, Galway Clinic, Doughiska, Royal College of Surgeons in Ireland and the University of Galway, Affiliated Teaching Hospitals, H91 HHT0 Galway, Ireland

**Keywords:** aortic stenosis, aortic regurgitation, aortopathy, bicuspid aortic valve calcification, cardiovascular computed tomography, transcatheter aortic valve implantation, photon-counting CT and 4D CT, artificial intelligence, machine learning, deep learning

## Abstract

Bicuspid Aortic Valve (BAV) disease is recognized as the most common congenital heart condition and is frequently associated with complex valvular and aortic disorders. Cardiovascular computed tomography (CT) has become essential for diagnosing BAV, planning procedures, and evaluating patients after treatment. This is largely due to CT’s high spatial resolution and its ability to perform volume imaging effectively. This review provides an up-to-date overview of the increasing role of cardiovascular CT in the management of bicuspid aortic valve (BAV). It covers various aspects, including BAV morphology, optimal sizing for transcatheter aortic valve replacement (TAVR), and post-procedural monitoring. We highlight significant innovations, such as supra-annular sizing techniques and artificial intelligence (AI)-guided analysis, that position CT at the nexus of anatomy, function, and targeted treatment. Additionally, we address controversies concerning inconsistencies in sizing algorithms, recent classification challenges, and radiation exposure. Future development areas include AI predictive tools, radiomic phenotyping, and CT-guided precision medicine. This synthesis aims to provide clinicians and researchers with a high-level guide to the clinical integration of cardiovascular CT and its future in the BAV population. This review provides the most current, comprehensive synthesis on the pivotal role of cardiovascular CT in BAV management, offering a roadmap for integrating advanced imaging into clinical practice and guiding future research priorities.

## 1. Introduction

**Figure 1:**
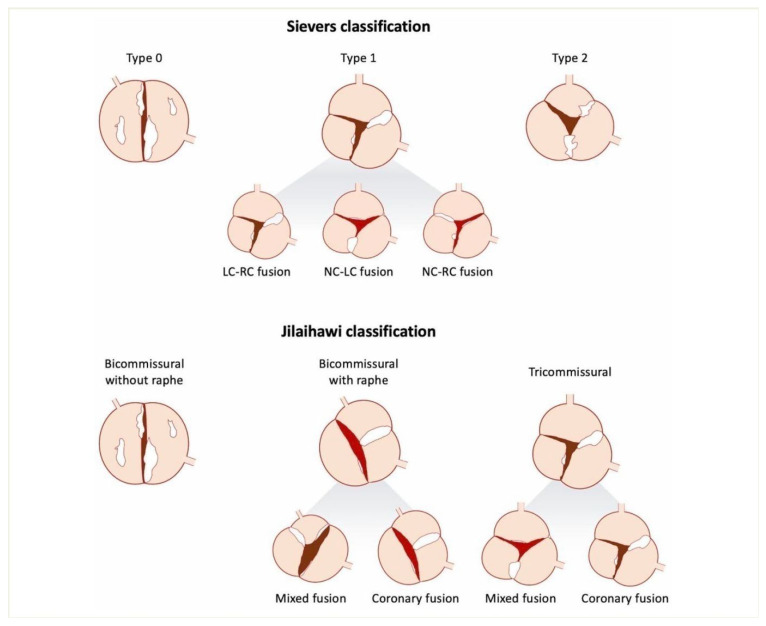
Sievers and Jilaihawi’s classifications of the bicuspid aortic valve.

Bicuspid aortic valve (BAV) disease is the most widespread type of congenital heart disease, with a prevalence of 0.5–2 percent of the general population. It has several structural and functional impairments. Due to the diversity of this anatomy, including raphe morphology, annular eccentricity, and other features, diagnosis, risk stratification, and intervention are challenging. The novel Jilaihawi classification of the BAV concerning TAVR is targeted at the cusp fusion’s presence and structure (Figure 1) [1]. It focuses on two planes, i.e., the interrelation between the prosthesis and the aortic valvular complex, at the basal leaflet plane and commissural plane. This categorization further divides the morphology of the aorta into three subcategories, i.e., bicommissural raphe, bicommissural non-raphe, and tricommissural bicuspid. Premature valve dysfunction, ascending aortic aneurysms, and challenging calcification deposits are issues that make traditional imaging insufficient in many cases [2,3].

Multidetector cardiovascular computed tomography (CT) has become a pillar in the management of BAV. It was initially applied to quantify the annulus and delineate calcium, but has more recently been used for functional analysis, procedural planning for transcatheter aortic valve replacement (TAVR), post-implantation surveillance, and the detection of complications such as paravalvular leak or prosthesis thrombosis. All the new developments, such as 3-dimensional reconstruction, low-dose acquisition protocols, and CT-based AI programs, have significantly improved the accuracy of diagnosis and therapeutic outcomes.

**Figure 2:**
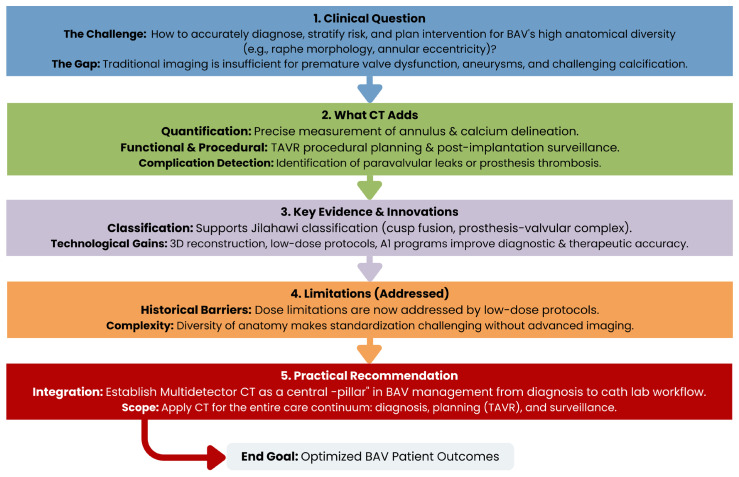
Overview of Cardiovascular CT in Bicuspid aortic valve (BAV) management.

In this review, we provide an up-to-date summary of the use of CT in the management of patients with BAV, from data acquisition to clinical workflow integration in catheterization laboratories. It explores emerging technologies poised to significantly impact clinical workflows and identifies crucial research directions necessary to achieve precision diagnostics and interventions in this complex patient population (Figure 2).

### 1.1. Literature Search and Review Methodology

To provide a comprehensive analysis of the role of Cardiovascular CT in managing Bicuspid Aortic Valve (BAV), we conducted a structured narrative review of the available literature.

To achieve our goal, we conducted a Narrative Synthesis of all relevant evidence, drawing on findings from primary research, clinical guidelines, and technology assessments.

We searched a wide range of primary electronic literature databases, including PubMed/MEDLINE, Scopus, and Google Scholar. The search strategy for our literature review focused on publications from 2019 through 2025 to ensure that our review captured the most current clinical methods and technological advancements (e.g., AI and Photon-Counting CT). A backward “snowball” method was then used to expand the number of relevant studies identified. We expanded on our search results by manually reviewing the references listed within each article we found through our electronic searches. These references may have included other relevant studies that were missed by our initial search.

The search strategy combined controlled vocabulary (MeSH terms) and free-text keywords, modified to match the syntax of each database.

- PubMed/MEDLINE: Specific Boolean strings were created using: (“Bicuspid Aortic Valve” [MeSH] OR “BAV”) AND (“Multidetector Computed Tomography” [MeSH] OR “Cardiac Imaging Techniques” [MeSH]).
- Scopus and Google Scholar: Additional broader keyword combinations were developed for Scopus and Google Scholar to identify emerging technologies and procedural applications; examples include: (“ECG-gated computed tomography” AND “BAV”), (“MSCT” OR “MDCT” AND “BAV”), and (“Transcatheter aortic valve implantation” AND (“artificial intelligence” OR “machine learning” OR “deep learning”)).
- Refine: To ensure the included studies link pathophysiology to intervention, additional specific terms were added to the refinement; examples include “Aortic Stenosis,” “Aortic Regurgitation,” and procedural acronyms like “TAVI/TAVR”.

### 1.2. Study Selection and Eligibility Criteria

The relevance of studies was evaluated through a multi-step process applied to each study considered for inclusion in this systematic review. First, the titles and abstracts of all identified studies were manually reviewed by the three authors (O.S., M.A.J., and CA). Second, the full texts of all potentially relevant studies identified during the initial screening were examined. Third, any disagreements between the review authors about which studies to include or exclude were resolved by consensus. To address potential discrepancies concerning the use of secondary literature, the authors clearly distinguished between the primary evidence base and the contextual background, as follows:
**Category****Inclusion Criteria****Role in Review****Primary Research**Original studies utilizing cardiovascular CT for diagnosis, morphological characterization, or procedural planning in confirmed BAV patients.Formed the core evidence base for diagnostic accuracy and outcomes.**Secondary Literature**Major society guidelines, consensus statements, and high-quality systematic reviews/meta-analyses.Used to establish current standards of care and clinical context.**Outcomes Data**Studies reporting diagnostic accuracy, prognostic value, or therapeutic planning outcomes (e.g., TAVR sizing).Used for comparative analysis of sizing methodologies.**Language**English language literature only.N/A

**Exclusion Criteria:** Excluded from this review were all non-English language articles, duplicate publications, and those that included only case reports with limited broader clinical significance. Studies in which cardiovascular CT was not the primary imaging modality being evaluated were also excluded; this exclusion ensured that the review would remain focused on the unique features of CT as an imaging modality.

### 1.3. Data Synthesis and Organization

The extracted data will be categorized under key thematic areas:

- Physiological and Pathological Characterization: Anatomical evaluation of BAV morphology and calcium burden.
- Diagnostic Accuracy: Effectiveness of CT compared to other modalities.
- Interventional Planning: Use of imaging for sizing and simulation for surgical (SAVR) or transcatheter (TAVR) procedures.
- Emerging Technologies: Incorporation of AI, radiomics, and photon-counting CT.

We combine findings from original research studies and comprehensive review articles to synthesize the current evidence and identify areas for future research. We evaluated quality based on whether the studies were relevant to the review objectives and their potential bias, ensuring the analysis reflects strong clinical evidence.

### 1.4. Contemporary Landscape of BAV Disease and Imaging Needs

BAV disease has a very high prevalence, affecting 0.5–2 percent of the global population, making it the most common congenital heart disease. Additionally, it is highly heterogeneous in structure and has a broad clinical spectrum beyond valvulopathy. It also shows enormous variability in the pattern of raphe fusion, which includes the associated risk of significant aortic dilation, aneurysm formation, and dissection. Conventional imaging methods, especially echocardiography, often fall short in providing detailed morphological descriptions, particularly for characterizing subtle raphe systems, quantifying asymmetric calcium deposits, and assessing the overall extent of involvement of the ascending aorta.

Today, the management of BAV is based on a nuanced understanding of its atypical pathophysiology. For example, the risk of aortic complications and the predisposition to significant obstruction depend directly on the localization and extent of cusp fusion. Similarly, morphological subtypes, defined by the presence/absence of a raphe, the depth of leaflet calcification, and the extent of annular eccentricity, have profound prognostic and procedural implications, particularly when planning an intervention [4,5,6,7]. Multimodal imaging plays a central role [8], albeit with multidetector cardiovascular computed tomography (CT), which improves the granularity and homogeneity of BAV assessment [9,10].

### 1.5. Principles of Cardiovascular CT in BAV

**Figure 3:**
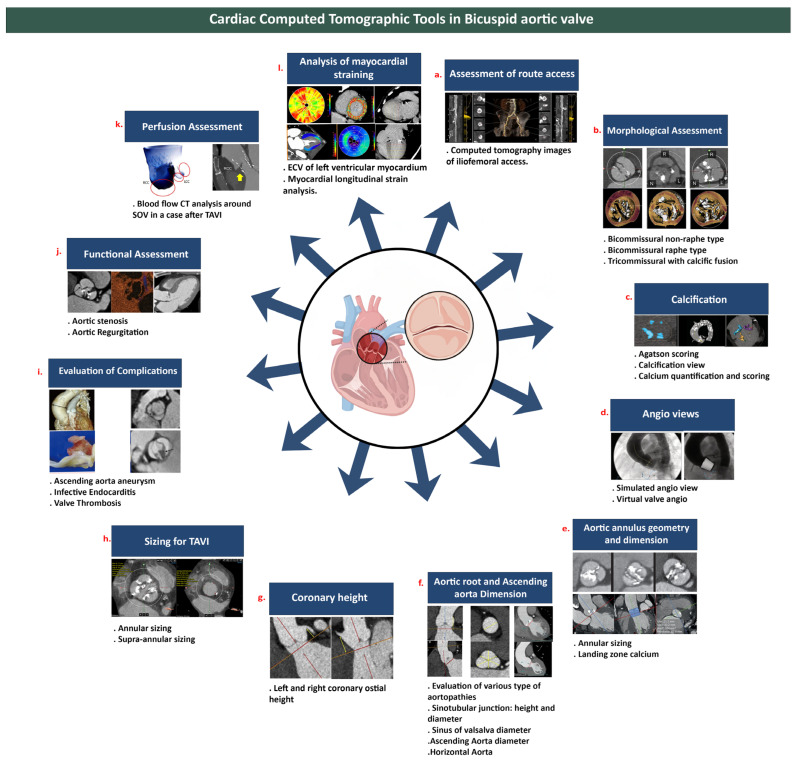
Cardiovascular Computed Tomography (CT) imaging tools in the bicuspid aortic valve. (**a**) Assessment of Route Access; (**b**) Morphological assessment of BAV; (**c**) Calcium quantification; (**d**) MDCT provides aortic annular sizing; (**e**) Assessment of the aortic root and ascending aorta; (**f**) Evaluation of coronary ostia and anomalous origin; (**g**) cardiovascular CT in sizing for TAVI showing BAV imaging through annular and supra-annular sizing; (**h**) Angio stimulation showing Simulated and Virtual Angio view; (**i**) Evaluation of complications BAV bicuspid aortic valve; (**j**) Functional assessment of BAV including aortic stenosis and aortic regurgitation; (**k**) Perfusion Assessment; (**l**) Analysis of myocardial straining; MDCT multidetector computed tomography, TAVI transcatheter aortic valve implantation, BAV bicuspid aortic valve.

The technological breakthroughs in spatial and temporal resolution that allow the superior visualization of the morphology of the leaflets, calcium burden [11,12], and aortopathy have made cardiovascular CT one of the most significant tools for evaluating BAV disease in depth and with detailed characterization over the recent years. Compared to other imaging modalities, cardiovascular CT has a broader field of vision, contrast resolution to characterize the aortic root and ascending aorta [13,14], and the measurement of annular and supra-annular dimensions, functional, and perfusion assessment, which are of the utmost importance during transcatheter interventions (Figure 3).

### 1.6. Aortic Valve Planimetry

Aortic valve planimetry is typically performed with the valve fully open, approximately 50 ms following the R wave, where the fully reconstructed peak systolic phase (50–150 ms) provides an optimal image for anatomical evaluation. While there is generally good agreement between multidetector CT (MDCT) and transesophageal echocardiography (TEE) for aortic valve area (AVA) measurements (r = 0.99 vs. r = 0.74) [15,16], CT demonstrates superior utility in cases where heavy leaflet calcification causes acoustic shadowing that obscures the valve orifice on echocardiography.

It is critical, however, to contextualize AVA measurements obtained via CT planimetry against standard Doppler-derived values. Studies indicate that CT-derived AVA, whether obtained through direct planimetry or utilizing the CT-derived left ventricular outflow tract (LVOT) area in the continuity equation, tends to be larger than the functional AVA estimated by TEE [15,17]. This discrepancy arises because CT measures the anatomical orifice area, whereas echocardiography measures the effective orifice area (vena contracta), which is hemodynamically smaller [18]. Consequently, to align with the hemodynamic severity of stenosis, some investigators have proposed adopting a higher cutoff of 1.2 cm^2^ for defining severe aortic stenosis when using CT planimetry, as opposed to the standard echocardiographic cutoff of 1.0 cm^2^ [15,18,19]. Clinicians should exercise caution when applying this threshold, as it is a methodology-specific adjustment rather than a universally applicable guideline modification, particularly when discrepancies exist among imaging modalities.

### 1.7. Cusp Calcification

Cardiovascular CT has also become a valuable complementary tool, particularly for assessing AV calcification (AVC) as it strongly indicates AS severity and prognosis, with sex-specific thresholds. The Agatston method quantifies AVC using ECG-gated CT, with severity thresholds of 1300 AU and 2000 AU for females and males, respectively [20]. Using multidetector CT, Gollmann-Tepeköylü and colleagues reported that BAV valves had higher calcification volumes, particularly in the non-coronary cusp [21]. Cardiovascular CT has also been shown to be more effective in distinguishing between bicuspid and tricuspid aortic valves, particularly when accounting for valvular calcification. Kim et al. reported that cardiovascular CT significantly outperformed TTE in sensitivity (*p* < 0.001), negative predictive value (*p* < 0.001), and accuracy (*p* = 0.003) [22].

### 1.8. Aortic Regurgitation

Aortic Regurgitation (AR) is more frequent in young patients with BAV than in those with AS. In younger patients, AR commonly arises because of prolapsing cusps, valvular destruction brought about by infective endocarditis, or complications of valve surgery. In adults, AR might be functionally acquired due to ascending aorta dilatation. BAV and degenerative AR are the most common causes of primary AR. Moderate to severe AS or AR is an important independent predictor of adverse cardiac events [23]. CCT is an excellent adjunct to the assessment of the mechanism of AR and measurement of the regurgitant aortic orifice area, and complements TEE by allowing accurate measurement of the ascending aorta, aortic root, and the annulus, all of which influence the indication and planning of the surgical intervention [24].

### 1.9. CT-Based Prediction of Aortic Valve Repair in BAV

Cardiovascular CT can be used to assess valve phenotype and symmetry, particularly commissural orientation, which is useful for determining repair viability and longevity. Detailed evaluation of the morphology of the leaflets, calcification, and geometric height can also be done with CT, and substantial calcification or leaflet retraction are considered the warning signs of an increased risk of failure after repair. In addition to this, the CT of the root and annulus should be evaluated, and asymmetry or excessive dilation of the root and annulus should be a reason to decrease the possibility of a permanent repair. The extensive morphological description of CT images aids the surgeon in selecting the most appropriate surgical modality and in determining whether repair or replacement of the damaged area is warranted in each case [24].

### 1.10. Aortopathy in BAV

Bicuspid aortopathy occurs in approximately 35–50% of patients with BAV [25]. Patients with BAV have greater ascending aorta and aortic sinus diameters in comparison to TAV, with defective elasticity resulting in increased risk of aortic dilatation [26,27]. The main diagnostic method for assessing BAV patients is TEE, which can delineate the size of the ascending aorta and aortic root, yet cannot assess the complete ascending and descending aorta. The gold standards in assessing dimensions of the aorta at different levels are CT and magnetic resonance imaging. Patients with BAV with a diameter of the aorta of ≥55 mm are advised to receive surgical aortic repair, while those with a diameter of ≥50 mm but with concomitant risk factors like aortic coarctation, systemic hypertension, family history of dissection, or an increase in aortic diameter at 0.3 mm/year should be evaluated for surgical repair of the aorta [26]. A cross-sectional area: height ratio > 10 cm^2^/m has been reported to be associated with a higher risk of type A dissection, measured at the tubular ascending aorta or the level of the sinus of Valsalva [26,28]. Bicuspid aortopathy makes dissection and rupture risk a concern when balloon valvuloplasty or balloon-expandable valve implantation is carried out.

Remarkably, recent CT-based protocols employ pre- and post-contrast imaging sequences. Therefore, the ability to analyze coronary ostia, anomalous origins, and other vascular structures can be done in a single sitting. The utilitarian scope of CT has also expanded: current methods allow semi-quantitative assessment of aortic regurgitation or stenosis and, using multiphase reconstruction, determination of annular changes during the cardiac cycle [29]. Specifically, dose-modulation algorithms and iterative reconstruction software are now available in updated versions that have reduced cumulative radiation dose, a key historical limitation that has impeded the use of CT in younger patients with BAV.

### 1.11. Morphological Classification and Emerging Challenges

BAV disease is complex to diagnose, as it exhibits substantial anatomical diversity. The historic standard of classification by Sievers and Schmidtke [4] is based on the number of raphes and the patterns of cusp fusion to determine the subtypes. However, recent studies indicate that it is irrelevant to interventional and functional aspects. As a case in point, ambiguous or incomplete raphe and massive asymmetric calcification, which influence device selection and procedural strategy, may confound echocardiographic and surgical subclassification.

Cardiovascular CT has evolved into multiplanar cardiac imaging, which provides greater fidelity, three-dimensional mapping of leaflet morphology and raphe anatomy, and direct guidance for risk stratification and procedural path [30,31,32]. Surprisingly, the annular eccentricity and the cusp imbalance are more associated with the procedure’s outcome and complications (such as paravalvular leak (PVL) or device migration) based on measurements on CT, despite the classical subtypes. However, interobserver variability remains, and no single CT-based classification is widely agreed upon, underscoring a significant unmet need to standardize platforms and studies.

**Figure 4:**
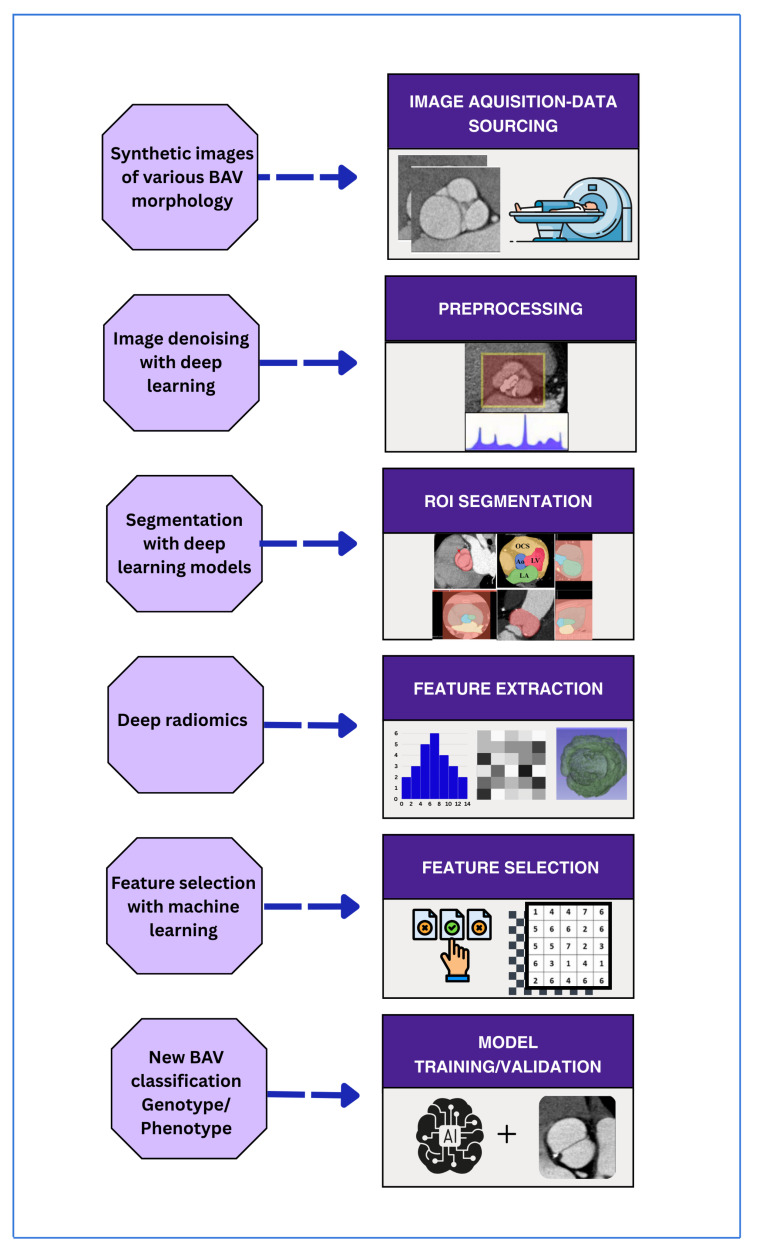
Integration of AI and Radiomics for developing BAV taxonomic application.

Recent recommendations suggest presenting hybrid schemes with a functional assessment, including stenosis and calcification burden, alongside aortic phenotypes (root, ascending, arch) using more specific morphological measures. Development of artificial intelligence (AI) and radiomics will further develop the taxonomic application of BAV through the phenotyping that is automated and reproducible phenotyping using CT, and this will ultimately inform the genotype-imaging characteristics and personalized management approach (Figure 4).

## 2. Integration of CTCA and CT-FFR in Pre-Procedural CT in BAV

### 2.1. Established Evidence in Standard Aortic Stenosis

Substantial evidence supporting Standard Aortic Stenosis comes from the use of computed tomography coronary angiography (CTCA) and CT-derived fractional flow reserve (CT-FFR) as non-invasive methods for assessing coronary artery disease (CAD) in patients with severe aortic stenosis. Studies (such as CAST-FFR [33] and FUTURE-AS [34]) and numerous single and multi-center studies [35,36,37] support the reasonable diagnostic sensitivity (range 73.9–86.4%) of CTCA and CT-FFR, as well as their excellent negative predictive values. Thus, CTCA and CT-FFR are effective gatekeepers that reduce the number of unnecessary invasive coronary angiograms (ICAs) and simplify patient referral paths [34,36,37]. The addition of machine learning (ML) to CT-FFR analysis also enhances its specificity and real-world applicability; it provides an excellent alternative to conventional pre-procedural invasive assessments [35].

### 2.2. Exploratory Status and Challenges in BAV Anatomy

Although significant advancements have been made in the use of CTCA and CT-FFR for pre-procedural evaluation of aortic stenosis, both remain primarily in the experimental stage in BAV populations. Clinical confidence regarding the use of these technologies is currently primarily based upon data obtained from tricuspid aortic stenosis populations, as opposed to being directly supported by data generated specifically within BAV populations [33,34]. Studies such as CAST-FFR and FUTURE-AS have demonstrated that CT-derived physiological assessment is valuable for the diagnosis of standard aortic stenosis; however, these studies have traditionally excluded patients with complex congenital heart disease and/or have not stratified outcomes by valve morphology [33,34,36]. As such, the inference that the high levels of diagnostic sensitivity and negative predictive values observed with tricuspid anatomy can be applied directly to BAV populations requires rigorous validation.

Transferring these technologies to BAV anatomy will present unique challenges due to anatomic and functional differences that are critical to imaging performance.

- Imaging Calcifications: BAV has greater and more asymmetric leaflet calcification than tricuspid valves [21], and this calcification burden results in increased artifact from both beam hardening and blooming that can hide the coronary ostia and result in decreased sensitivity (false positives) for CTCA-based assessments of coronary stenosis [33,35].
- Functional Hemodynamic: The function assessments by CT-FFR rely on computational fluid dynamics (CFD), assuming normal laminar flow conditions. Due to the presence of a raphe and annulus ellipticity in BAV, eccentric turbulent flow jets occur [35], and it is currently unknown if these flow disturbances would decrease the accuracy of current CT-FFR algorithms that have not been validated in the complex hemodynamic environment of bicuspid aortopathy [37].

**Figure 5:**
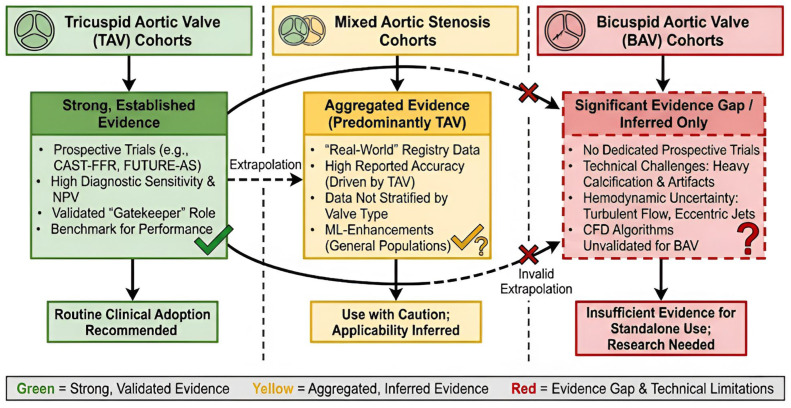
Evidence maps illustrate diagnostic utility for CTCA and CT-FFR in Aortic valve disease by valve morphology.

Furthermore, while optimized protocols utilizing pharmacological vasodilation have improved feasibility in general cohorts, safety concerns persist regarding their use in BAV patients who may present with concomitant aortic aneurysms or severe ventricular dysfunction [33,35]. While CTCA and CT-FFR hold the potential to provide a safe, less invasive alternative to invasive angiography, current evidence is insufficient to recommend their routine adoption in BAV. Future research must move beyond extrapolation, utilizing dedicated prospective studies to verify diagnostic accuracy specifically within this high-risk population, ensuring that the streamlining of patient care does not come at the cost of diagnostic precision (Figure 5) [37].

## 3. CT for Interventional Procedural Planning in BAV

### 3.1. The Foundation: Annular vs. Supra-Annular Sizing

**Figure 6:**
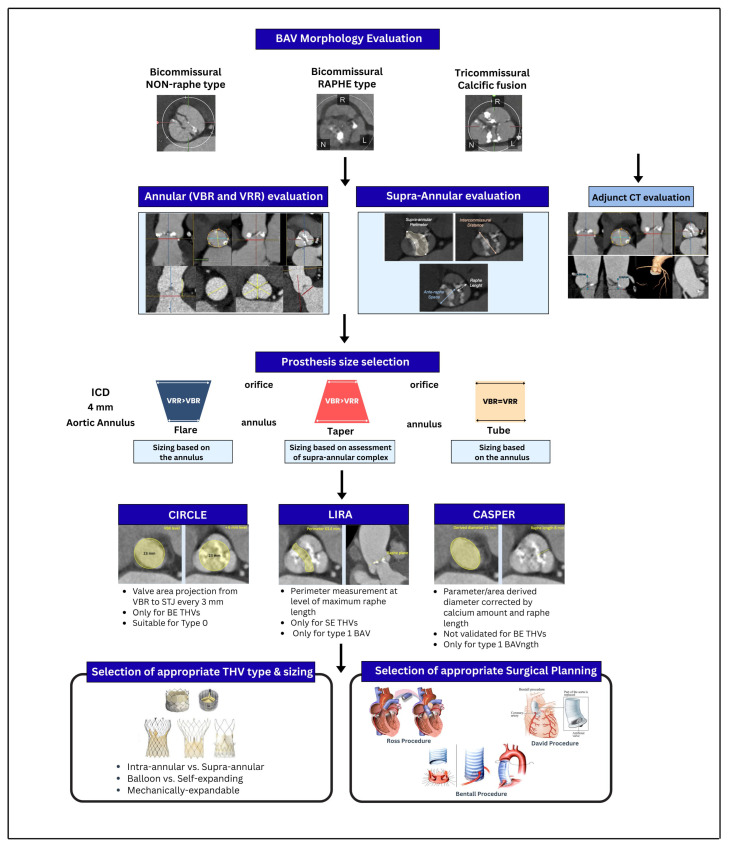
Illustration of Cardiovascular CT-Guided Sizing Strategies for TAVR/SAVR in BAV.

Cardiovascular CT has revolutionized the planning of transcatheter aortic valve replacement (TAVR) by providing high-fidelity assessment of annular dimensions, calcium distribution, and coronary ostial heights [13,14]. The choice of sizing strategy, annular versus supra-annular, is critical in BAV due to the frequency of tapered anatomies and eccentric orifices (Figure 6).

#### 3.1.1. Annular Sizing (Standard of Care)

Area-based and perimeter-based sizing during systolic peak at the level of the virtual basal ring are the gold standards for most balloon-expandable valve (BEV) sizing [22,23], as they emphasize the importance of annular sealing to prevent PVL. However, when considering a BAV, strictly annular sizing may underestimate the required prosthesis size due to significant tapering of the anatomy, or if the functional orifice is determined by the supra-annular fusion.

#### 3.1.2. Supra-Annular Sizing (Exploratory/Complex Anatomy)

The ICD method measures the inter-commissural distance approximately 4 mm above the annulus. This measurement is particularly important to measure for self-expanding (SE) platforms in tapered, raphe-type bicuspid aortic valves (BAVs) due to the supra-annular apparatus constraining the placement of the SE platform as opposed to the annulus itself [38].

### 3.2. Advanced Sizing Algorithms: When and Why to Use

**Figure 7:**
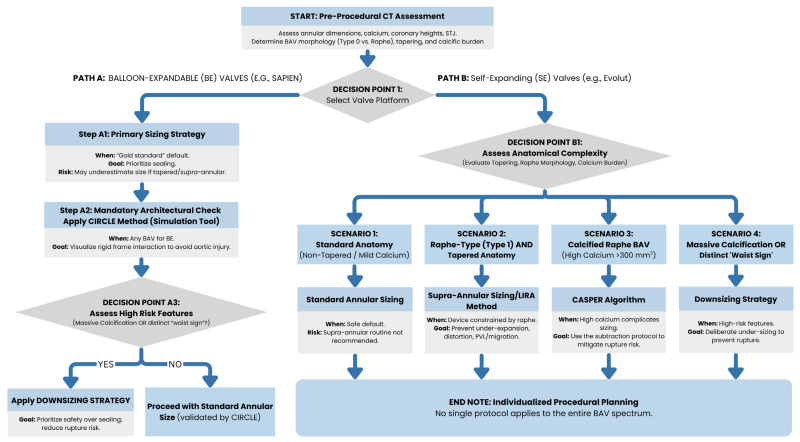
Algorithmic approach to CT-guided TAVI sizing in Bicuspid aortic valve anatomy. The decision-making process begins with a comprehensive CT assessment and valve platform selection, then diverges into distinct pathways for balloon-expandable (BE) and self-expandable (SE) valves. Each path incorporates anatomical evaluation and specific sizing strategies (Standard, LIRA, CASPER, CIRCLE & Downsizing) tailored to mitigate procedural risk, i.e., paravalvular peak, annular rupture, and device distortion.

Several device-specific and anatomy-specific algorithms have also been developed to assist with the sizing challenges presented by many complex BAV phenotypes (Figure 7).

#### 3.2.1. LIRA (Level of Implantation at the Raphe)

Designed specifically for self-expanding valves (i.e., Evolut) in Raphe-Type BAV. The LIRA method identifies a “neo-annulus” at the highest point of the raphe’s protrusion. Therefore, by sizing at this supra-annular plane versus the basal ring, the LIRA method attempts to prevent both under-expansion and deformation of the valve. The evidence supports that the LIRA method is safe and reduces PVL in certain types of raphe type anatomy [39,40].

#### 3.2.2. CASPER (Calcium Algorithm Sizing for Bicuspid Evaluation with Raphe)

A step-by-step approach to self-expanding valves that considers both calcium burden and raphe morphology. Unlike LIRA, CASPER is quantitative and adjusts the target valve size based on the calcium score (calcium > 300 mm^3^) and the raphe-to-annulus ratio, and it also provides an additional subtractive protocol (0–2.5 mm) to reduce the risk of annular rupture in calcified anatomy [41].

#### 3.2.3. CIRCLE Method

Developed for balloon-expandable valves (SAPIEN 3/Ultra). This method projects a virtual cylinder (matching the prosthesis diameter) from the annulus to the sinotubular junction. It is used to visualize the interaction between the rigid valve frame and the native anatomy at multiple levels, ensuring that the chosen size accommodates the “waist” of the BAV without risking aortic injury [42].

### 3.3. The Role of Downsizing

In cases where there are large amounts of calcification, or when a definite “waist sign” can be seen on balloon sizing, a Downsizing Strategy will be utilized with the purpose of prioritizing safety over successful sealing of the valve. This approach, validated in trials such as the Chinese Downsize Study and TAILOR-TAVR, involves selecting a valve size smaller than the CT-measured annulus would indicate. Early results indicate that this method is noninferior to standard sizing and may reduce the incidence of annular rupture and the need for a new pacemaker, without increasing the incidence of moderate-to-severe PVL [43,44].

### 3.4. Evidence Summary and Clinical Consensus

**Figure 8:**
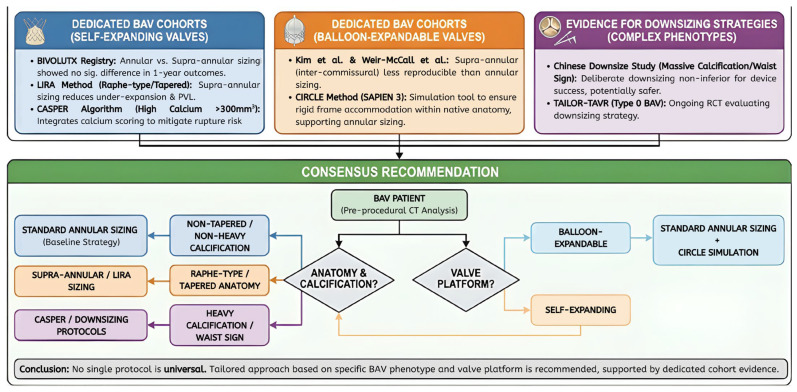
Evidence Map: Cohort-specific findings and consensus recommendations.

Recent trials have sought to validate these diverse strategies. The BIVOLUTX Registry compared annular versus supra-annular sizing for SE valves and found no significant difference in clinical outcomes (mortality, PVL, pacemaker rates) at one year, suggesting that while supra-annular sizing is feasible, annular sizing remains a safe default for most anatomies [45,46,47]. Although the LIRA and CASPER trials suggest the need for supra-annular awareness in raphe-heavy phenotypes to optimize hemodynamic performance [39,40,41,48], other trials emphasize that annular sizing is still the safest and most predictable method for the general population and caution against routine supra-annular sizing without evidence of tapering (Figure 8) [22,49,50].

As a result, there is no single protocol to apply to all types of bicuspid aortic valve (BAV) disease. Each patient’s procedural plan should be tailored to the individual, using the CIRCLE method for balloon-expandable devices to ensure optimal anatomical fit, and using LIRA or CASPER for self-expanding valves when raphe and/or calcium burden may interfere with proper valve expansion. (Table 1).

## 4. Innovation Spotlight: Downsizing, Trials, and Artificial Intelligence

### 4.1. Procedural Innovation: Sizing Trials and the Downsizing Strategy

Current procedural innovations in BAV management are primarily driven by prospective randomized clinical trials evaluating the impact of specific sizing techniques (as outlined in Table 2). The “Downsizing” method of sizing has become a well-established technique for overcoming anatomically challenging cases and is particularly useful in cases with massive calcification, a stiffened raphe, or a “waist sign” observed during balloon predilation. Data from an early study, the Chinese Downsize Study, demonstrates that deliberately downsizing the selected valve size to be one size less than the calculated perimeter of the annulus will decrease the rate of annular rupture and new permanent pacemaker placement without significantly increasing the incidence of moderate to severe PVL at one year compared to standard annular sizing [44]. The ongoing TAILOR-TAVR trial will also validate this approach in Type 0 BAV cases and assist in establishing a standardized protocol for anatomically challenging phenotypes [43].

Conversely, the BIVOLUTX registry compared the outcomes of annular and supra-annular sizing for self-expanding valves. Results demonstrated that annular and supra-annular sizing resulted in equivalent one-year clinical outcomes (mortality, PVL, pacemaker placement) [45,46,47]. Therefore, although supra-annular sizing (and the LIRA method), as described in previous studies [39,40,48], assures circumferential deployment in raphe-type valves, the remainder of patients may safely utilize standard annular sizing. Overall, recent evidence supports a personalized approach, utilizing annular sizing for standard anatomy, and reserving downsizing or supra-annular sizing for patients with either high-calcium or tapered phenotypes [22,50].

### 4.2. Artificial Intelligence: Differentiating Statistical Learning from Deep Learning

The integration of Artificial Intelligence (AI) into cardiovascular CT extends beyond merely automating procedures. It is therefore essential to differentiate between traditional statistical methods and new machine learning (ML) architectures.

#### 4.2.1. Logistic Regression (LR) vs. Advanced ML

Traditional Risk Scores (such as STS and EuroSCORE II) are based on logistic regression, which remains the foundation of clinical prediction due to its interpretability and transparency. Systematic Reviews and Meta-Analyses, including those conducted by Zaka et al., have demonstrated that advanced ML architectures (Random Forests, XGBoost, Support Vector Machines, etc.) may achieve higher discriminative accuracy on complex, nonlinear datasets than well-tuned LR Models. This has not been consistently demonstrated using standard tabular clinical data [51].

#### 4.2.2. Deep Learning (DL) and the “Black Box” Challenge

In Image-Based Tasks (Automated Segmentation of the Aortic Valve and Coronary Tree), Advanced AI, specifically Convolutional Neural Networks (CNNs), will outperform traditional methods. DL Algorithms can isolate the aortic root and quantify the calcium burden with pixel-level resolution and precision that far exceed those of manual thresholding. However, the “Black Box” problem of CNNs, i.e., the fact that the internal decision-making process is often opaque, represents a major barrier to clinical adoption since clinicians need to be able to trust a risk prediction but cannot see the transparent coefficient explaining why a patient is classified as high-risk [52,53].

### 4.3. Radiomics: Technical Capabilities and Reproducibility Crises

Radiomics is an important new approach that shifts from visual interpretation to high-dimensional data mining. The extraction of numerous quantitative features (e.g., texture, shape, skewness, entropy) from standard CT scans may enable the identification of phenotypic subgroups in patients that correlate with their genetic predisposition or treatment outcomes [54,55]. The potential clinical applications of radiomics are currently limited by significant technical barriers to realizing its full theoretical potential:

#### 4.3.1. Segmentation Variability

Radiomics is very sensitive to the area you select as your Region of Interest (ROI). Small changes in how you define the aortic valve manually or semi-manually could result in large differences in the radiomic feature values, which could limit the reliability of results [55,56].

#### 4.3.2. Lack of Standardization

Unlike Hounsfield Units, there are no standard radiomic features that have been defined for all vendors of CT scanners or kernels of reconstruction.

#### 4.3.3. Validation Gaps

Most of the BAV radiomics studies are single-center retrospective studies without external validation; therefore, they are at high risk of “overfitting” to their own dataset and will not be able to predict results in other populations with BAV [54].

The future of research should focus on creating a common approach to extracting radiomic features, and on developing “Explainable AI” to increase the confidence of clinicians in using radiomics [55].

An innovative clinical paradigm is the integration of artificial intelligence (AI) and radiomics into modern preoperative diagnostic protocols for bicuspid aortic valve (BAV) disease, which can transform automated, reproducible, and non-interventional treatment. It begins with the acquisition of standardized volumetric cardiovascular computed tomography (CT) images. It is followed by the use of deep-learning-based segmentation algorithms, which automatically identify and outline the aortic valve, root, and other pertinent anatomical landmarks, resulting in a clinical-grade level of accuracy and low dependence on human expertise [52,56]. The outcome is a radiomic data set that includes features of texture, shape, and tissue composition. This is analyzed using machine-learning classification techniques to more accurately stratify the BAV taxonomy than traditional visual inspection and to reveal phenotypic clusters predictive of underlying genotypes and clinical outcomes [56].

When using AIs to automate the measurement of annular and supra-annular dimensions, interobserver variability decreases, and the ability to select a prosthesis commensurate with the patient improves, thereby enhancing the sizing of transcatheter heart valves (THV) [52,57]. Complex algorithms also estimate the optimal THV size and simulate device-tissue interactions, which may ultimately increase the procedure’s success rate and reduce potentially fatal complications such as paravalvular leaks [57]. Imaging, clinical, and procedural data integrated into AI models can achieve higher accuracy than predictions of short- and long-term outcomes after both surgical aortic valve replacement (SAVR) and transcatheter aortic valve replacement (TAVR), thereby assisting in real-time decision-making and personalization [51,53].

This diagnostic workup can be supplemented with CT coronary angiography (CTCA) and CT-derived fractional flow reserve (CTFFR), which can be used in the pre-procedural assessment to provide fully comprehensive hemodynamic data. CTCA provides systematic anatomical mapping of the coronary arterial tree. However, CTFFR, using either computational fluid dynamics or AI, detects functionally significant lesions and provides anatomic and hemodynamic information from a single CT scan [58,59,60]. This comprehensive approach enables aggressive risk stratification and allows clinicians to identify patients who may be candidates for revascularization procedures, while also enabling reclassification of management in as many as 21% of patients compared with anatomic assessment alone [59].

**Figure 9:**
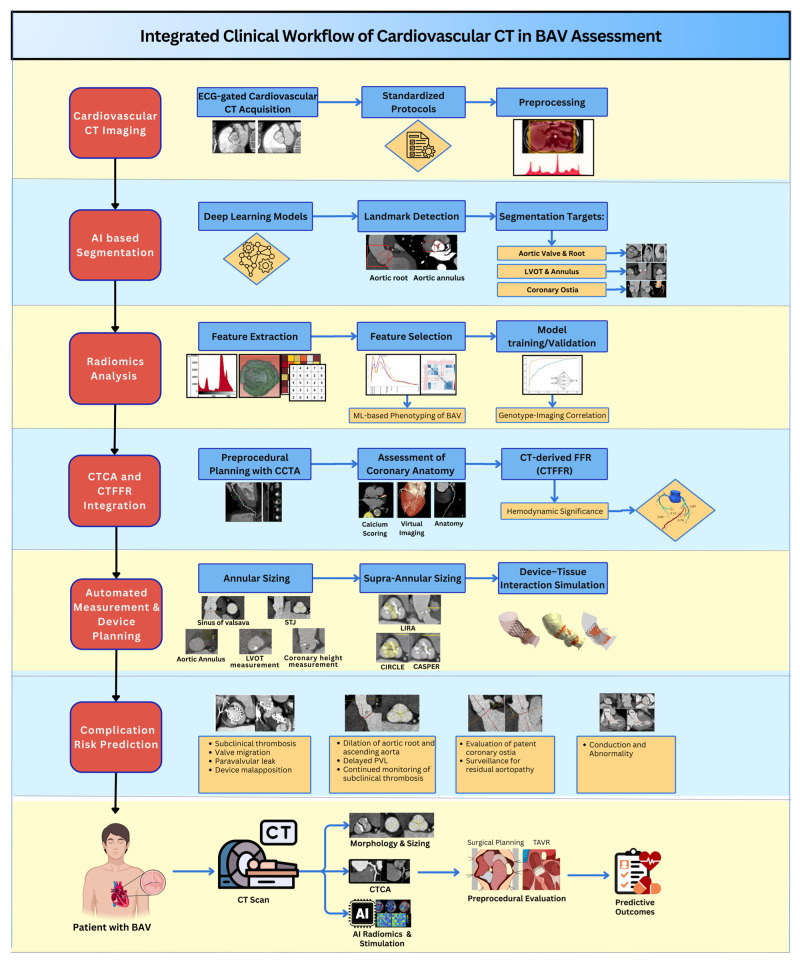
Integrated clinical workflow of Cardiovascular CT in BAV assessment as a one-stop modality.

In practice, a patient with BAV referred for an intervention might undergo a single CT scan that automatically assesses valve morphology, coronary anatomy, and coronary lesion hemodynamics. Simultaneously, AI algorithms evaluate the risk of adverse events associated with SAVR and TAVR, thereby directing therapy based on individualized risk profiles. This efficient, evidence-based workflow streamlines BAV classification, optimizes the size of implanted devices, and enhances the efficiency and accuracy of preprocedural planning, ultimately advancing precision medicine in valvular heart disease (Figure 9) [52,56,58].

#### 4.3.4. Post-Procedural CT Surveillance

**Figure 10:**
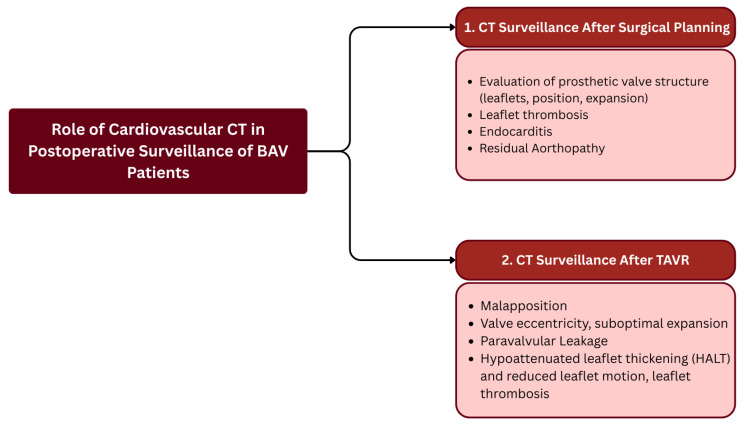
Post-Procedural Cardiovascular CT Surveillance Workflow.

Cardiovascular CT plays a central role in the postoperative management of patients with BAV by assessing complications and guiding the evaluation of surgical outcomes after valve repair or replacement. MDCT will also enable sensitive detection of silent adverse events, including prosthesis thrombosis, valve migration [61,62,63], or paravalvular leak (PVL) [64]. The heart valve (aortic root) and the ascending aorta ought to be measured quantitatively since progressive dilation is a significant risk factor after the procedure. Advanced ECG-gated imaging also confirms leaflet movement, prosthesis expansion, and minor device malapposition or subclinical thrombosis [62,63,65]. Under the guidance of the nature of complex anatomy or residual aortopathy, MDCT follow-up should be given to patients to measure patent coronary ostia (Figure 10). Multiphase, low-dose, and high-resolution protocols are gaining importance as a means of balancing diagnostic value against radiation and contrast load.

#### 4.3.5. Limitations, Unmet Needs, and Controversies

**Figure 11:**
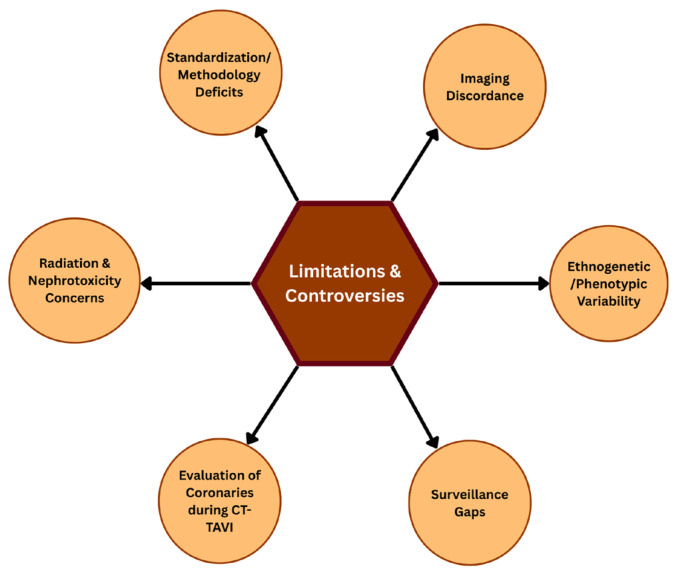
Cardiovascular CT: Limitations and controversies.

Despite its unparalleled anatomical information, cardiovascular CT in BAV remains controversial [13,14] (Figure 11). The complexity of the procedure is also evident in the debate over the most effective method for sizing TAVR [22,38]. Various strategies, such as annular, supra-annular, LIRA, and downsizing, have been used at other centers [22,39,40,41,42,43,44,48,50]. Recent registry-based data can provide information about the efficacy of various sizing methodologies [44,45,46,47]. However, long-term follow-up is still needed to evaluate outcomes, including paravalvular leak and valve performance [44,47]. The problem of interobserver variability in CT-derived parameters for valve sizing raises concerns about standardizing the imaging protocol, which may influence procedural success and patient outcomes [22,49,50].

There is discordance between cardiovascular computed tomography (MDCT) and echocardiography in determining the degree of aortic stenosis [15,16]. Compared with echocardiography, MDCT is highly accurate, as it can provide detailed three-dimensional images of valves, particularly in cases of severe calcification, whereas echocardiography underestimates stenosis [21,22]. Cutoffs and the measurement process are not standardized across modalities, hindering the harmonization of a standard measure [17,18,19]. Imaging modalities should be harmonized, and protocols standardized with cutoff values to improve the accuracy of diagnosing aortic stenosis in patients [19].

In patients with BAV, especially among the younger population, radiation exposure and nephrotoxicity are significant concerns that need to be addressed [29]. In addition to the low-dose imaging regimens, repeated radiation exposure can have long-term health implications, particularly in patients with BAV [66,67]. It requires frequent imaging to monitor aortic size and valve performance [26,27]. The newer contrast agents are safer, but they still expose one to the risk of renal injury, especially when there is pre-existing renal dysfunction. It is necessary to consider alternative imaging modalities and employ nephroprotective measures when obtaining the required diagnostic data for the management of BAV [68,69].

Further research is needed to elucidate the multifaceted interactions between genetics and the environment in BAV-related aortopathy across diverse ethnicities [25,28]. Awareness of these variations can help in making a more personalized risk assessment and management of patients with the BAV disease [44]. Additionally, investigating non-invasive imaging modalities that reduce radiation exposure and contrast agent requirements without compromising diagnostic capabilities can enhance long-term outcomes and quality of life [66,67,68].

The current landscape underscores the value of CTCA and CT-FFR in routine TAVR workup for standard aortic stenosis while highlighting significant evidence gaps and operational challenges in complex anatomies, making clear that their widespread adoption should be guided by ongoing research, clinical context, and a nuanced appreciation of patient heterogeneity [33,34,35,36,37,58,59,60]. The actual application of imaging follow-up protocols in aortic disease in real-world settings is not straightforward, and breaches in surveillance and subsequent late identification of complications have been reported [61,62,63,64,65]. Patient-related factors that affect adherence include lack of awareness and financial constraints, whereas healthcare system-related issues include poor communication between providers. To address these gaps, patient education must be enhanced, procedures simplified, and tracking improved. Improved synchronization with providers and harmonization can enhance compliance with guidelines and patient outcomes [8].

## 5. Future Directions: Imaging in the Precision Era

**Figure 12:**
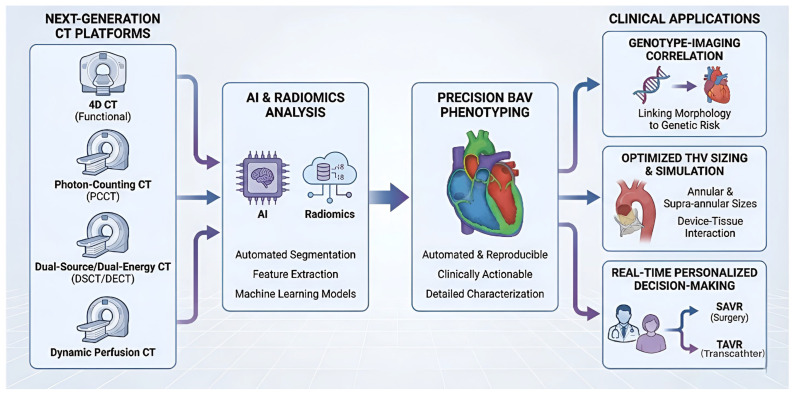
Future Direction: AI and advanced Cardiovascular CT modalities.

Existing trends in cardiovascular CT of bicuspid aortic valve (BAV) indicate that precision, automation, and integration will be the characteristics of the field in the future (Figure 12). Artificial intelligence (AI), radiomics, and next-generation CT platforms (e.g., 4D CT, photon-counting CT (PCCT), dual-source/dual-energy CT (DSCT/DECT), and dynamic perfusion CT) are converging to revolutionize BAV assessment and management (Figure 10). All these innovations have the potential to make CT-based phenotyping more automated, reproducible, and clinically actionable than ever, with implications that extend far beyond genotype-imaging correlation, transcatheter heart valve sizing (both annular and supra-annular), and real-time, patient-specific decision-making between surgical (SAVR) and transcatheter (TAVR) intervention [55].

### 5.1. D CT Imaging

4D CTs, which now adequately depict the beating heart and aortic valve in dynamic three-dimensional views, are vital to recent advances in cardiac imaging. They include an integrated explanation of valve structure and dynamic function over the cardiac cycle [70]. 4D CT is a valuable tool for understanding the hemodynamic implications of BAVs, particularly stenosis or regurgitation. It helps identify valve gradients and determine valve failure rates more accurately than traditional static imaging. This imaging modality also aids in patient selection for TAVI/TAVR procedures, enabling better differentiation between annular size and calcification pattern. This improved accuracy in sizing and placement of devices reduces the risk of complications during procedures [71,72]. In recent work, Fikani and coworkers employed this novel BAV image analysis technique based on 4D MCCT and software advancements. The method enabled a simpler evaluation of BAV morphology, nonfunctional commissural features, and commissural orientation, while allowing a 3D dynamic examination of the AA throughout the cardiac cycle. Aortic root aspects vary significantly amongst the many forms of BAV, which comprise a spectrum of morphology [73].

### 5.2. Photon-Counting CT

Recently, photon-counting detector CT has become clinically available and has eliminated several key intrinsic limitations of traditional CT. PCD CT, rather than scintillation detectors, uses semiconductor detectors in which X-ray photons are converted into electrical signals before being measured individually. These detectors provide ultra-high-resolution imaging with a section thickness of 0.2 mm, thereby effectively eliminating beam-hardening and blooming artifacts, improving spatial resolution and contrast, and reducing radiation dose. Higher resolution facilitates visualization of small, microscopic anatomical features, such as leaflet thickness and tiny calcifications, which are particularly important for young patients who are likely to require multiple sessions. In general, PCD CT significantly reduces radiation exposure, thereby reducing the need for long-term monitoring, particularly for conditions such as BAV [66,67,68]. Current evidence indicates that PCD CT offers distinct benefits for imaging patients before and after TAVR. The high spatial resolution enables detailed visualization of the coronary arteries, the aortic valve, the aortic root, and potential vascular access routes. Furthermore, PCD CT’s spectrum reconstruction features, such as Virtual monoenergetic images (VMIs) and metal artifact removal, may improve leaflet and stent imaging [68]. Photon-counting CT may replace conventional CT in the routine scanning of BAV patients, considering improved diagnostic sensitivity and enhanced patient safety due to the higher reduction in radiation [68].

### 5.3. Dynamic Perfusion CT

Dynamic perfusion CT (DPCT) is a diagnostic tool used to assess flow through the heart and aortic valve after contrast agent administration. It provides crucial information on hemodynamic effects, including the flow profile across the stenotic valve, which is essential for assessing the functional severity of valve obstruction. DPCT is vital in treatment selection for patients with cardiac ailments like BAV, where symptoms do not correlate with stenosis revealed on imaging. Combining anatomical and functional information enables a more comprehensive assessment, potentially aiding decision-making before procedures such as TAVR or surgical valve repair [69,74].

Artificial intelligence (AI), radiomics, and enhanced computed tomography (CT) are converging to support precision phenotyping, genotype-imaging association, and data-driven personalized therapeutic decision-making in BAV, thus transforming care delivery according to empirical, one-size-fits-all models to evidence-based individualized management. Such integration will improve diagnostic and prognostic accuracy and generate new research opportunities to elucidate the molecular and genetic determinants of BAV and, ultimately, improve patient outcomes by enabling earlier intervention with better-matched devices and more tailored follow-up recommendations. However, the clinical implementation rate will depend on rigorous validation, clarity of the regulatory pathway, and clinical integration. However, the trend is toward a more automated, data-driven, and patient-focused future of cardiovascular care.

## 6. Conclusions

Cardiovascular CT has now become the foundation of BAV imaging. It has become the benchmark against which to frame interventions and risk-stratify patients, particularly those with anatomically complex or heavily calcified valves. Follow-up CT imaging after the procedure should be risk-stratified for the patient, and modern protocols should balance image completeness with the need to reduce radiation dose and contrast. Other modes of change, such as downsizing, supra-annular-based sizing, and AI-optimized analytics, are reshaping device selection, procedure planning, and outcomes; further multicenter validation is required. The next BAV management boundary crossing will occur through multidisciplinary collaboration, robust data from international registries, and consistent, evidence-based strategies grounded in CT-based processes. Ultimately, the evidence gap regarding BAV will need to be closed by using artificial intelligence and next-generation technologies, such as photon-counting CT, in conjunction with large-scale, multicenter registries to transition the field from empirical observations to precision-based, individualized management of patients with BAV.

## Figures and Tables

**Table 1:** Comparative Overview of CT-Guided Sizing Strategies in BAV TAVI.

Strategy	Primary Device Applicability	Key Anatomical Indication (When/Why)	Key Evidence/Outcome
**Standard Annular Sizing**	BE & SE	Default strategy. Best for Type 0 BAV or mild calcification, where anatomy is non-tapered.	BIVOLUTX [45,46,47]: Comparison showed similar safety/efficacy to supra-annular sizing. Remains the most reproducible standard.
**Supra-Annular (ICD)/LIRA**	SE	Raphe-Type BAV (Type 1). Used when the “functional” annulus is higher due to fused leaflets, or to avoid device constraint in tapered aortas.	LIRA Studies [39,40,48]: 100% device success in initial cohorts; reduced risk of PVL and migration in raphe-type valves.
**CASPER Algorithm**	SE	Calcified Raphe BAV. Used when a high calcium burden (>300 mm^3^) complicates sizing. Adjust size to prevent rupture.	Petronio et al. [41]: Validated algorithm integrating calcium score and raphe length to customize downsizing.
**CIRCLE Method**	BE	Any BAV (esp. Type 0/1). Simulation tool to check if the rigid BE valve frame fits the anatomy from annulus to STJ without injury.	Blackman et al. [42]: Consensus method for SAPIEN 3/Ultra to visualize device-tissue interaction at multiple levels.
**Downsizing (e.g., Hangzhou)**	BE & SE	High Calcium/Waist Sign. Deliberate under-sizing to prevent annular rupture in rigid, heavily calcified anatomy.	TAILOR-TAVR [43], Chinese Downsize [44]: Non-inferior device success; favourable for reducing rupture and pacemaker rates.

Abbreviations: BE = Balloon-Expandable; SE = Self-Expanding; BAV = Bicuspid Aortic Valve; ICD = Inter-Commissural Distance; PVL = Paravalvular Leak; STJ = Sinotubular Junction.

**Table 2:** Clinical trials (2019–2025) on sizing strategies of transcatheter heart valves in bicuspid aortic valve disease.

Trial/Study	Sizing Strategy	Methodology	Key Outcomes	Conclusion
**TAILOR-TAVR** (NCT05511792)	**Downsizing** (Hangzhou Solution) vs. Standard Annular	RCT in Type 0 BAV. Self-expanding valve. Valve one size smaller if “waist sign” and mild regurgitation after pre-dilatation.	Results awaited. Preliminary data suggests higher device success and fewer pacemakers in the downsizing arm.	Downsizing may improve clinical outcomes in challenging anatomies [43].
**BIVOLUTX Registry** (NCT03495050)	**Annular vs. Combined** (Annular + Supra-annular)	149 patients (Evolut). Compared outcomes by sizing strategy using CT-based measurements	No significant difference in valve performance, mortality, PVL, or PPM at 1 year.	Clinical outcomes were similar regardless of methodology; annular sizing remains safe [45,46,47].
**LIRA Method Studies** (incl. SUBLIME)	**Supra-annular** (Level of Implantation at Raphe)	Cohorts (Single centres/registries). Sizing based on the minimum CT perimeter at the raphe versus the annulus.	100% device success in initial series; reduced valve migration and moderate/severe PVL.	Supra-annular sizing appears safe and effective in select raphe-type BAV [39,40,48].
**Chinese Downsize Study**	**Annular** **vs.** **Downsize**	Cohort of 293 (95 BAV). Outcomes at 1 year comparing device success, gradient, and PVL.	Device success: 82% (downsizing) vs. 83.3% (annular); similar gradients and PVL rates.	The downsizing strategy is non-inferior and safe, particularly in high-calcium anatomy [44].
**Weir-McCall et al.**	**Annular vs. Supra-annular** (ICD-based)	44 BAV patients (SAPIEN 3). Compared to CT-measured annulus vs. Intercommissural Diameter (ICD).	Supra-annular (ICD) sizing was less reproducible; annular sizing agreed best with implanted size.	Annular sizing is more consistent; routine supra-annular sizing is not recommended [22,49,50].

## Data Availability

The original contributions presented in this study are included in the article. Further inquiries can be directed to the corresponding author.
